# Molecular Mechanisms of Retinal Pigment Epithelium Dysfunction in Age-Related Macular Degeneration

**DOI:** 10.3390/ijms222212298

**Published:** 2021-11-14

**Authors:** Jongmin Kim, Yeo Jin Lee, Jae Yon Won

**Affiliations:** 1 Department of Mechanical Engineering, Pohang University of Science and Technology (POSTECH), Pohang 37673, Korea; mandarinbear@postech.ac.kr; 2 Department of Ophthalmology and Visual Science, Eunpyeong St. Mary’s Hospital, The Catholic University of Korea, Seoul 03312, Korea; yeojin@nate.com; 3 Catholic Institute for Visual Science, College of Medicine, The Catholic University of Korea, Seoul 14662, Korea

**Keywords:** age-related macular degeneration, retinal pigment epithelium

## Abstract

The retinal pigment epithelium (RPE), situated upon Bruch’s membrane, plays multiple roles in the ocular system by interacting with photoreceptors and. Therefore, dysfunction of the RPE causes diseases related to vision loss, such as age-related macular degeneration (AMD). Despite AMD being a global cause of blindness, the pathogenesis remains unclear. Understanding the pathogenesis of AMD is the first step for its prevention and treatment. This review summarizes the common pathways of RPE dysfunction and their effect in AMD. Potential treatment strategies for AMD based on targeting the RPE have also been discussed.

## 1. Introduction

Retinal pigment epithelium (RPE) cells form a monolayer on Bruch’s membrane and play various roles in the retina and choroid, maintaining homeostasis of the ocular system. The pigmented monolayer absorbs the entered light and alleviates oxidative stress. Tight junctions in the RPE control the molecular transportation by forming a blood-retinal-barrier. The RPE also removes molecular wastes from photoreceptors to support the visual cycle. Furthermore, the RPE secretes several types of growth factors and provides ocular immunity [1].

Due to their crucial role, dysfunction in the RPE causes diseases related to human vision, including age-related macular degeneration (AMD). AMD is the one of the most common causes of blindness in developed countries, especially in the elderly population, and the number of patients is expected to increase with an increase in population aging [2,3]. Moreover, the patients are expected to over 288 million by 2040 due to the lack of the therapies for dry AMD, the most prevalent form [4,5]. Unfortunately, the exact disease pathogenesis is still unknown. Several factors, including inherited genetic variations, oxidative stress, ethnicity, obesity, smoking, and hypertension, are reported as risk factors; however, aging is considered the most important one [6]. Despite the cause of AMD being unknown, the disease is affected by various human body systems including chronic low-grade inflammation, imbalance of the systemic immunity, and local ocular factors, such microglia, ganglion and Muller cells [7,8]. Among them, one of the major factors is the dysfunction of the RPE. In addition, changes in the RPE and its microenvironment are reported in AMD patients.

This review introduces the functions and structure of the RPE, describes its role in AMD development, and discusses potential treatment strategies that involve targeting the RPE.

## 2. AMD

**Figure 1:**
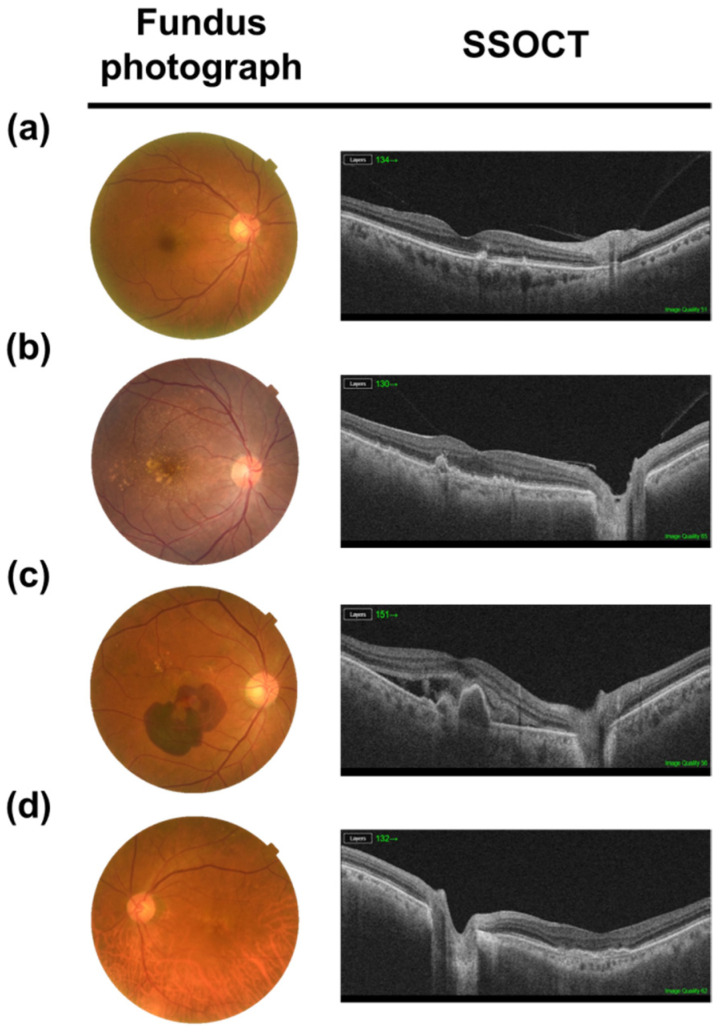
Multimodal image of age-related macular degeneration (AMD). Color fundus photograph and swept source optical coherence tomography (SS-OCT) images show the feature of early, intermediated AMD, neovascular AMD and geographic atrophy (**a**,**b**). Non-neovascular AMD (dry AMD): small and intermediate soft drusen. (**c**) Neovacular AMD (wet AMD): submacular hemorrhage, subretinal fluid and pigment epithelial detachement. (**d**) Geographic atrophy: retinal pigment epithelial pigment and photoreceptor atrophy at fovea.

AMD is an age-related ocular dysfunction, which causes the central vision loss. It is classified as either dry or wet type. Wet or neovascular AMD is characterized by the invasion of blood vessel from choroid into the sub-retinal/-RPE space, which manifests as fluid release or hemorrhage (intraretinal, subretinal, or subretinal pigment epithelium), retinal pigment epithelium detachment, hard exudate, or subretinal fibrous scar tissue. These symptoms destruct the ocular system and lead to vision loss [9]. Depending on their stages, the disease also be classified into early, intermediate, or advanced AMD [10] (Figure 1). The Age-Related Eye Disease Study categorized disease based on several factors—density and size of drusen, location and area of RPE distruption, and choroidal neovascularization (CNV) [11]. Pigment irregularities in the retina and drusen and the accumulated extracellular debris between RPE and choroid are presented in early AMD. The drusen could be classified by size as small (smaller than 63 µm), intermediate (between 63 µm and 125 µm), and large (larger than 125 µm) [12]. Intermediate and large drusen were observed in intermediate AMD patients and indicate a higher risk of late or advanced AMD [11]. Half of the patients with extensive drusen progress to geographic atrophy and develop blindness or neovascularization within 5 years [13,14,15]. Unfortunately, these severe conditions do not have a cure. Anti-angiogenic factors have been tested in neovascular AMD patients; however, they only delay disease progression, and vision loss could occur depending on the initial retinal tissue state, such as disruption, scarring, and atrophy.

## 3. Structure of the RPE

The RPE forms pigmented monolayer with hexagonal cells. Their size and shape are diverse depending on the features of retinal anatomy [16,17,18,19,20,21]. Their diameter ranges from 14 μm in the fovea to 60 μm in the peripheral retina. Their height ranges from 10–15 μm in the fovea, while it is 7.5 μm in the peripheral retina [22]. In addition, the fovea has a higher density of RPE cells than other regions [23]. The density of RPE cells in the peripheral retina decreases with age, however, the inward migration of peripheral RPE preserves density of RPE in fovea [24]. The RPE plays a multifunctional role to maintain retinal homeostasis. It interacts with overlaying photoreceptors by direct contact via microvilli on their apical surface [1]. Each RPE connects 30–40 photoreceptors, and the microvilli envelop the photoreceptor outer segments (POS) to facilitate molecular transportation [24]. Complex infoldings of the basal surface of the RPE also allow molecular transportation in the choroid.

The structure of RPE also reveals this apical-basal polarity. Most of the components, including nucleus and melano-lipofuscin granules, are located on the basal side; however, melanosomes are located on the apical side [19,25]. The directionality of molecular movements depends on ion pumps, polarly distributed, and channels on the apical and basal sides. For instance, for the maintenance of ionic homeostasis in the sub-retina, nutrients are transported to the sub-retina or molecular wastes are removed from photoreceptors.

## 4. Role of the RPE

### 4.1. Blood-Retinal-Barrier

The molecular control system called outer blood-retinal-barrier (oBRB) is formed via tight junction of RPE together with Bruch’s membrane [26,27]. The cells are connected via tight junction protein, such as ZO-1, with adjacent RPE cells to seal the interconnected regions. These junctions are crucial for oBRB formation, blocking the free movement of toxins, large molecules, blood-borne products, and even water. This barrier system makes intercellular molecular transportation 10-fold more efficient than pericellular molecular transportation [28]. However, retina-derived diffusible factors could damage the system. Among them, vascular endothelial growth factor (VEGF) is the most widely studied molecule; the studies reported the breaking down of the oBRB in diabetic edema and in *in vitro* experiments [29,30,31].

### 4.2. Protection from Oxidative Stress

The high metabolism rate induces oxidative stress in the retina. The RPE pigments absorb reflected and scattered light, which not only enhances the image quality but also protects the retina against oxidative damage due to increased local oxygen tension, high metabolism, continuous exposure to light, and the photo-oxidation of lipofuscin [19]. Especially, the melanin in the RPE reduces the photo-oxidation of lipofuscin by filtering the harmful light [32,33]. In addition, it removes reactive oxygen species (ROS) [34]. The density of melanin is higher in the center of the retina, with the highest density in the fovea. The intrinsic antioxidant property of the RPE can be attributed to the presence of enzymes, including superoxide dismutase (SOD), catalase, and cytochrome P450 monooxygenase, and non-enzymatic molecules, such as thiol, ascorbate, thioredoxin, β-carotene, and glutathione [35,36].

### 4.3. Transport of Nutrients, Wastes, and Water

#### 4.3.1. Transport from Blood to Photoreceptors

The oBRB controls the molecular transportation in the ocular system. Nutrients, including fatty acids, ascorbate, and glucose, and fatty acids, are transported from the choroid to photoreceptors via transporters on the RPE membrane.

GLUT1 (glucose transporter 1) and GLUT3 transport glucose passively [37]. The fundamental glucose transportation is conducted by GLUT3; however, depending on the metabolic situation, GLUT1 is used for inducible glucose transportation. GLUT2 and GLUT5 are recently discovered glucose transporters found on cultured RPE cells [38]. Sodium-dependent transportation is used to transport ascorbic acid, a scavenger of superoxide radicals [39]. The transportation of fatty acids occurs in a concentra-tion-dependent manner [1]. Docosahexaenoic acid (DHA) is important for visual func-tion since it is the main component of the photoreceptor membrane and because the membrane is continuously consumed in the POS due to the clearance functions of RPE via phagocytosis [40]. Furthermore, DHA precursor is transformed into the anti-oxidant antioxidant neuroprotectin D1.

#### 4.3.2. Transport from Subretinal Area to Blood

Several ions and water are transported from the subretinal area to the choroid. Metabolic activities of photoreceptors produce a large amount of water. Water movement from the vitreous humor induces pressure on the retina. Therefore, continuous removal of the water is required, which is facilitated by Müller cells in the inner retina and RPE in the subretinal region [41]. In the RPE, the transportation of water depends on Cl- and K+ movements, and the removal of water enhances the adhesion force between the retina and RPE [42,43]. RPE is classified as a tight epithelium and has a 10-fold higher resistance to paracellular transportation than transcellular transportation, making it nearly impossible for water to pass via boundaries of cells; water is mainly transported through transcellular pathways via aquaporin-1 [44,45,46].

### 4.4. Phagocytosis of POS

The phagocytosis of POS by RPE is essential for the maintenance of photoreceptor excitability, recycling the nutrients and preventing the photo-oxidation of damaged POS. The high light exposure induces the accumulation of photo-damaged proteins and lipids in photoreceptors. Therefore, the constant renewal of POS is required to maintain photoreceptor excitability [47]. Especially, free radicals and photo-damaged molecules are accumulated in the tips of the POS. RPE eliminates shed POS, containing molecular waste, via phagocytosis.

### 4.5. Production and Secretion of Growth Factors

The RPE also secretes various cytokines and growth factors to maintain homeostasis of the ocular system that provide structural stability to maintain the supply and circulation of nutrients and the survival of photoreceptors. These factors include pigment epithelium-derived growth factor (PEDF), VEGF, lens epithelium-derived growth factor (LEDGF), platelet-derived growth factor (PDGF), ciliary neurotrophic factor (CNTF), fibroblast growth factor (FGF), tissue inhibitor of metalloprotease (TIMP), insulin-like growth factor-1 (IGF-1), and members of the interleukin family [48,49,50,51,52,53,54]. PEDF is an anti-angiogenic factor secreted to the apical layer to maintain the fenestrated structure of the choriocapillaris. TGF-β regulates inflammation and extracellular matrix secretion. In addition, TGF-β and TIMP together regulate the turnover in the extracellular matrix. While PDGF regulates cell growth and healing, photoreceptors are protected by PEDF, CNTF, IGF-I, FGF, and LEDGF as neuroprotectant growth factors. VEGF is secreted from the basal side of the RPE and controls the permeability of the choriocapillaris. VEGF overexpression is key for choroidal neovascularization and is the main focus of wet AMD research [55].

### 4.6. Visual Cycle

The visual cycle is the conversion of the projected image data into electric signals and depends on the retinoid exchange between RPE and photoreceptors. The initial step of the cycle is light absorption by rhodopsin in the POS, composed of opsin, G-coupled receptors, and the chromophore 11-cis-retinal, and 11-cis-retinal is converted to all-trans-retinal [56]. The lack of *cis-trans*-isomerase in photoreceptors induces the metabolism of all-*trans*-retinal to all-*trans*-retinol. Then, the retinol is transported into RPE and re-isomerized to 11-*cis*-retinal via *cis-trans*-isomerase and re-transported into photoreceptors for subsequent visual cycles.

### 4.7. Immune Privilege

The RPE maintains immune privilege in the eye via the oBRB, immunosuppressive factors TGF β, interleukin 11, and interferon β, and complement proteins and regulators [57]. The oBRB forms a microenvironment that carefully regulates immune cell infiltration into the retina. In addition, Fas-ligand and Fas-expressing leukocytes induce apoptosis [58]. Furthermore, mass histocompatibility complex class I and II are expressed in the RPE and act as antigen-presenting cells in the ocular system [59]. Complement proteins and their related proteins, including complement 3 (C3), complement factor B (CFB), complement factor H (CFH), complement factor D (CFD), and complement factor I (CFI) are also synthesized in the RPE. In addition, the cells express complementary regulatory proteins such as membrane cofactor protein (MCP), decay accelerating factor, and CD59 on their membrane.

## 5. AMD Pathogenesis

While the exact pathogenesis of AMD is not fully understood, RPE dysfunction has a crucial role in both dry and wet AMD.

### 5.1. Complement Dysregulation in AMD

The complement system of innate immunity is essential for preventing inflammation. The eye is an immune-privileged organ, which can tolerate the introduction of antigens with its limited immune responses. The RPE is the primary driver source of complement activation in the retina. The constituents of the complement system are strictly regulated to small quantities in the eye [60]. This system could be activated via classical, mannose-binding lectin, and the alternative pathway. Among these systems, the AP is the major pathway related to AMD pathogenesis.

Inappropriate increases in complement activation are implicated in AMD pathogenesis [60,61,62,63]. Immunocytochemical analysis of drusen components and AMD lesions revealed a significant number of complement components, such as C3, C5, C9, complement factor F and H (CFF, CFH), and membrane attack complex (MAC) [64,65]. The AMD patients have elevated level of C3, C3d, Bb, and C5a [66].

The geographic atrophy (GA) is believed to occur usually because of drusen disturbing the transportation and removal of nutrients and wastes, respectively. This disturbance results in cell death in GA [61]. While the relationship between neovascular AMD and the impaired complement system is unknown, C3a, C5a, complement factor B, and MAC were shown to increase CNV lesions in a laser-induced CNV animal model by increasing the angiogenic factors secretion such as VEGF, TGF-β2, and β-FGF from the RPE [67,68].

The AMD is also affected via genetic variants of the complement system [69,70,71]. Genetic variations in CFB, C2, serpin peptidase inhibitor clade G member 1 (a complement component 1 inhibitor), and C3 increase the risk of AMD. The complement system may exacerbate the chronic local inflammation in AMD. C3a and C5a can stimulate the secretion of inflammatory cytokines including interleukin-1β, -6, -8, granulocyte-macrophage colony-stimulating factor, and MCP-1 from the RPE [72].

Oxidative stress could make the RPE more susceptible to complement-associated injury [73,74]. RPE cells under oxidative stress exhibit reduced expression of CD55 and CD59 and increased expression of CFH [75]. In human RPE cells, VEGF secretion is increased up to 100 times due to synergy between the complement cascade and oxidative stress [76].

### 5.2. Dysfunctional Mitochondria in RPE

Dysfunctional mitochondria in the RPE may be a critical cause of AMD pathogenesis [77,78,79]. Mitochondria mainly fulfill the demands of energy from cells by producing adenosine triphosphate (ATP) via oxidative phosphorylation, citric acid cycle, and β-oxidation. RPE also metabolizes fatty acids to synthesize β-hydroxybutyrate as an auxiliary source of energy [80]. RPE has abundant mitochondria to fulfill the energy needs for the outer retina [81].

Mitochondrial dysfunction leads to damaged respiration, which results in ROS accumulation [82]. Oxidative stress in mitochondria may aggravate the production of ROS leading to apoptosis. The study showed the mitochondrial-based AMD model via treatment of H2O2 to RPE for exhibiting mitochondrial DNA damage [83]. ROS overproduction could cause and result in significant mitochondrial DNA damage [84,85]. The intracellular ROS level is regulated by the antioxidant system, but an overwhelming ROS level leads to cell damage [86]. Although the mechanism of production of ROS in cells is not exactly clear, theories include cytochrome c interaction, damage to the SOD2 gene, and lipofuscin deposition [87,88,89].

SOD2, a primary antioxidant enzyme, protects the cell from damage due to oxidative stress by removing ROS [90]. In an SOD2-knockout mouse model, an increase in ROS level resulted in mitochondrial changes and RPE dysfunction [91]. ROS levels can increase due to interactions between the mitochondria and cytochrome c oxidase and the accumulations of lipofuscin in RPE cells [92,93].

### 5.3. Pathways of RPE Cell Death in AMD

**Figure 2:**
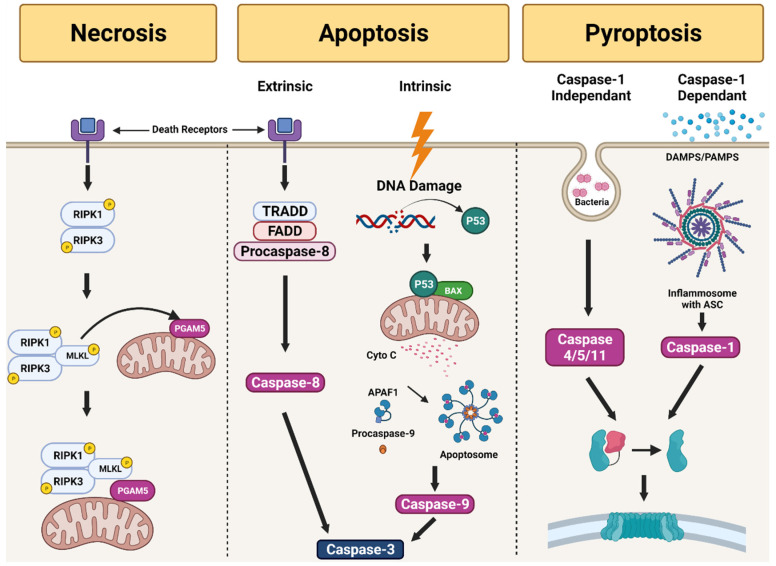
Overview of necrosis, apoptosis, and pyroptosis pathways involved in retinal pigment epithelium (RPE) cell death in age-related macular degeneration. In necrosis, the necrosome forms complex proteins that attach to the mitochondrial membrane, triggering cell death. Apoptosis can be activated through the intrinsic or extrinsic pathways, which eventually result in the activation of caspase 3 and then cell death. Pyroptosis can occur through caspase 1-dependent or independent pathways, which lead to the generation of an N-terminal fragment that induces cell death.

The death of photoreceptors and RPE occurs through apoptosis in AMD. However, necrosis and pyroptosis pathways have also been reported [94,95]. In the subsequent sections, we have concisely reviewed necrosis, apoptosis, and pyroptosis, the cell death pathways, to understand the mechanisms of RPE degeneration in AMD (Figure 2).

Necrosis is uncontrolled cell death induced by hypoxia or inflammation. This pathway is activated by an increase in diverse pro-inflammatory proteins, such as nuclear factor-κB, leading to the destruction of the cell membrane. The cell contents spilled in the pericellular space cause inflammation and tissue damage. The binding of the tumor necrosis factor (TNF) ligand to death receptors of cellular membrane and its trimerization initiates the necrosis pathway. This pathway is mostly controlled by receptor-interacting protein kinases (RIPs). Necrosome formation occurs after autophosphorylation of RIPK1 and RIPK3 in the absence of caspase 8. The necrosome phosphorylates mixed lineage kinase domain-like and recruits phosphoglycerate mutase 5, which attaches to the mitochondrial membrane to stimulate dynamin-related protein 1, resulting in death of the cell [94]. The involvement of the necrosis pathway in RPE cell death has been studied previously [96,97]. In these studies, the typical characteristics of necrosis included depletion of ATP and aggregation of RIP3 in ARPE 19 cells treated with H2O2 or tertbutyl hydroperoxide to induce oxidative stress. RPE cell death due to oxidative stress was prevented when RIPK3 was not activated [96]. Furthermore, RPE cells exposed to oxidative stress presented morphological features similar to necrosis such as swelling of the cells and loss of cell membrane integrity [97].

Apoptosis is the process including, inhibition of growing and dividing, eventually resulting in controlled death without leakage of its contents into the nearby environment. It is also called programmed cell death. The activation of a chain of cysteine-aspartic proteases known as caspases initiates the apoptosis. There are two types of caspases: initiator caspases and executioner caspases [98]. The damage of cell activates the initiator caspases (caspases 8 and 9) to induce the activation of executioner caspases (caspases 3, 6, and 7). This process results in DNA and nuclear fragmentation, cytoskeleton destruction, and the formation of apoptotic bodies. Apoptosis can be initiated through the intrinsic and extrinsic pathways. The intrinsic pathway involved in the mitochondria depends on factors secreted from the mitochondria. Stressors such as hypoxia, toxins, radiation, ROS, and viruses activate the intrinsic pathway [98]. The cellular damage leads to severe damage in DNA, which results in the suppression of anti-apoptotic factors and secretion of proapoptotic factors, like Bcl-2-associated X protein (Bax). Under Bax stimulation, the cytochrome C is released into the cytoplasm. Then, the apoptosome is formed via binding of cytochrome c with apoptotic protease activating factor 1 (Apaf1). The apoptosome then induce the activation of pro-caspase-9 and caspase-9. Among them, the caspase-9 induces the apoptosis by activating caspase-3 [98,99]. After binding of the TNF ligand to death receptors, the extrinsic pathway is activated. The death-inducing signaling complex is formed via activation of TNF-receptor 1-associated death domain and Fas-associated death domain. Then, the caspase-8 is activated to induce caspase-3 based apoptosis. The involvement of apoptosis in RPE degeneration is well known. Active caspase-3 was found in the RPE cells of a patient with GA [100]. Furthermore, Ho et al. reported that because c-Jun N-terminal kinases and p38 mitogen-activated protein kinase are necessary for Bax translocation to mitochondria, inhibitors of these suppressed the transposition of Bax to the mitochondria in oxidative stress [101]. In a recent study, exposure of RPE cells to the NOD-like receptor family pyrin domain containing 3 (NLRP3) inflammasome, which initiates inflammatory cell death, activated the apoptosis and pyroptosis pathways [102].

Pyroptosis is the process of programmed cell death with collateral damage through inflammation. This pathway can be activated by caspase 1 either independently or dependently. In a caspase 1-dependent pathway, inflammasomes play an important role. Inflammasomes are large multiprotein complexes formed in response to pathogen-associated molecular patterns and damage-associated molecular patterns. NOD-like receptor family proteins such as NLPR1, NLPR3, and NLPR4 of inflammasomes activate caspase-1 with ASC as the adapter protein. Caspase-1 then promotes the cleavage of the pro-pyroptotic factor gasdermin D, producing an N-terminal fragment that induces cell death. In case of the caspase-1-independent pathway, caspase-4/5 of human and caspase-11 of mouse promote the cleavage of gasdermin D to activate pyroptosis [95].

NLRP3 inflammasomes have been reported in GA and CNV lesions [103]. The proteolytic cleavage of caspase-3 (apoptotic pathway) and gasdermin D (pyroptotic pathway) was found in the RPE and choroid tissues of rats treated by intravitreal amyloid-beta injections [104]. Exposure of primary RPE cells to oxidative stress primed inflammasome formation, and the mechanism of the cell death was switched from apoptosis to pyroptosis [105]. There are some conflicting reports about the mechanisms of RPE cell death in AMD. Further research is required to better understand of these pathways.

### 5.4. Autophagy

Autophagy maintains cellular homeostasis by the lysosomal degradation of unused and damaged cellular components [106]. AMPK and mammalian target of rapamycin (mTOR) regulate autophagy as a promotor and an inhibitor, respectively. Autophagy is initiated by the formation of a phagophore from the endoplasmic reticulum. Phagophores stretch and enclose the cytoplasm and organelles to form a double-membrane autophagosome. Several autophagy-related proteins (ATGs) together with the LC3 conjugation system form mature autophagosomes with a closed bilayer membrane. After maturation, the autophagosome fuse with the lysosome to develop the autolysosome, which degrades waste material [106,107]. Autophagy in RPE cells usually occurs to maintain the homeostasis of RPE cells [108].

Although the exact role of autophagy in AMD remains unclear, impaired lysosomal degradation owing to the accumulation of lipofuscin is closely related to autophagy in AMD. Cathepsins are lysosomal proteases in the RPE. They degrade POS, forming lipid peroxidation end products and oxidized low-density lipoproteins in RPE cells [107]. Their accumulation induces RPE stress and activates lipofuscinogenesis [109]. Lipofuscin cannot be broken down by lysosomal enzymes and may augment oxidative stress in the RPE. It can decrease lysosomal cathepsin activity, which could result in the accumulation of autolysosomes, causing drusen.

Markers of autophagy have been discovered in the drusen of AMD donor tissue [110]. A decrease in autophagy occurs when RPE cells are chronically exposed to oxidative stress mediated by H_2_O_2_, but autophagic biomarkers increase when RPE cells are exposed to acute oxidative stress. Autophagy is generally activated in early AMD because of compensatory mechanisms increasing oxidative stress in the RPE; however, in the late stages of AMD, the autophagy pathway is unable to counter the large amount of damaged organelles and thus becomes dysfunctional [108]. Potential therapeutic strategies targeting autophagy may be useful. Therefore, further research into this pathway is warranted.

### 5.5. α. B Crystallins and RPE Crystallin in AMD

αA and αB crystallins are the main members of the small heat shock proteins (sHSP) family. sHSP help assemble cellular proteins, guide misfolded proteins, and prevent proteins from denaturing under external stress [111]. sHSP are involved in anti-apoptotic and proapoptotic pathways. αA crystallin is located largely in photoreceptors, astrocytes, and Müller cells, whereas αB crystallin is located predominantly in the RPE and localized to the mitochondria and Golgi apparatus [112].

In one study, the expression of αB crystallin was significantly increased in the RPE of patients with advanced AMD and drusen of patients with neovascular AMD and early atrophic AMD [113]. The expression of αB crystallin was also increased when RPE cells were exposed to oxidative stress induced by H2O2 [111]. αB crystallin stimulates VEGF and protects the protein against aggregation and unfolding [114]. This is possibly the cause of neovascular AMD developed through VEGF overproduction in the RPE. Other studies have also reported increased αB crystallin expression in angiogenesis and a significant reduction in VEGFA expression in αB crystallin-knockout mice [115,116].

Regarding the protective function of αB crystallin in the retina, αB crystallin prevents oxidative stress-mediated apoptotic cell death of the RPE. An increase in apoptotic activity was reported in a crystallin-knockout mouse model [117]. αB crystallin could potentially inhibit apoptosis via interaction with p53 to prevent its translocation to the mitochondria [118]. The increased production of αB crystallin also inhibits ROS activation to prevent apoptosis [119].

## 6. Treatment Targeting RPE in AMD

Patients with wet AMD are currently treated with anti-VEGF agents to achieve symptomatic relief. However, dry AMD has limited treatment options, such as lifestyle changes and vitamin supplements. Therapeutic strategies targeting RPE cells include the use of inhibitors of the complement pathway and visual cycle, neurotrophic factors, modulators of lipid metabolism, photobiomodulation (PBM) agents, and cell-based therapy. In the subsequent sections, we concisely review PBM and RPE transplantation methods targeting RPE in AMD.

### 6.1. PBM

PBM uses radiation in the visible to near-infrared spectrum (500–1000 nm) produced by laser or light-emitting diodes. Near-infrared radiation activates cellular functions by stimulating photoreceptors [120,121]. The therapeutic effect of PBM has been reported in animal models and patients with various retinal diseases, such as AMD [122,123], retinitis pigmentosa, and diabetic retinopathy [124].

#### 6.1.1. Mechanism of PBM

**Figure 3:**
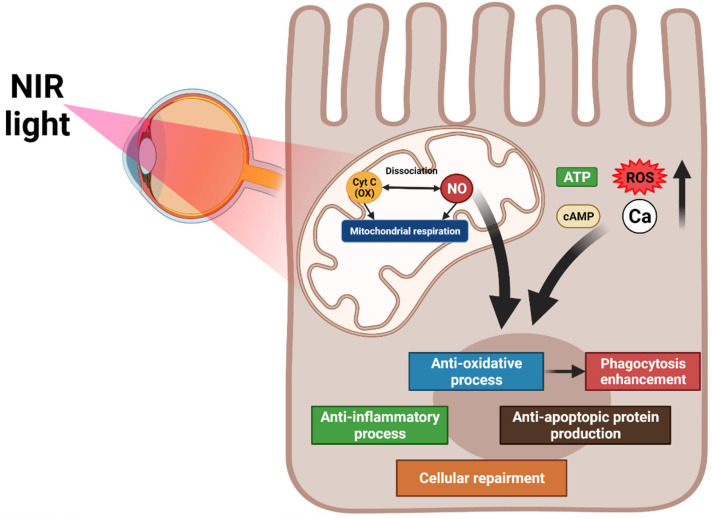
Overview of the mechanisms of photobiomodulation (PBM). PBM activates mitochondrial cytochrome oxidase C and increases mitochondrial respiration and nitric oxide dissociation. These processes elevate ATP, cAMP, reactive oxygen species, and intracellular calcium levels, promoting anti-inflammation, antioxidation, protein synthesis, anti-apoptosis, and cellular metabolism. The antioxidation effect of PBM increases RPE phagocytosis.

PBM targets the mitochondrial cytochrome oxidase C, which controls the oxygen and nitrite levels in tissues by directly activating mitochondrial respiration and indirectly increasing nitric oxide dissociation. A resultant increase in ATP, cAMP, ROS, and intracellular calcium levels promote anti-inflammation, antioxidation, protein synthesis, anti-apoptosis, and cellular metabolism. PBM inhibits oxidative stress, which increases RPE phagocytosis through the upregulation of phosphorylated Mer tyrosine kinase (MerTK) [125] (Figure 3). 

#### 6.1.2. Application of PBM at AMD

PBM prevents AMD pathogenesis through its effect on cellular oxidative stress, apoptosis, and inflammation. Furthermore, PBM removes drusen in dry AMD by increasing RPE phagocytosis. Lavey reported that near-infrared radiation increases ATP and nitric oxide levels in RPE cells, which presumably promotes mitochondrial oxidative phosphorylation through the non-binding of nitric oxide from cytochrome oxidase C [126]. Kokkinopoulos et al. reported that after near-infrared radiation light exposure increases the mitochondrial membrane potential and decreases C3d, TNF-α, and macrophages in an animal AMD model, demonstrating an improvement in mitochondrial function and reduction in RPE inflammation [127].

In one study, 348 eyes with dry and wet type AMD were treated with radiation from a 780 nm semiconductor laser diode, and 97% of patients with cataracts and 94% of patients without cataracts showed an average visual improvement and reductions in pigmentation and cystic drusen and improvements in metamorphopsia and dyschromatopsia were observed [122].

The TORPA II study demonstrated functional changes, such as enhancement of contrast sensitivity and visual acuity, and anatomical improvements, such as reduction in drusen volume and central drusen thickness, after PBM in patients with dry AMD [128].

### 6.2. RPE Cell Transplantation

Unlike for early AMD and wet AMD, no appropriate treatment exists for GA. However, cell-based therapy has recently been used to treat vision loss in late AMD, especially GA. Cell transplants might be used as a rescue or replacement therapy. Rescue therapy may preserve the function of dying tissue and also restore the function of dying cells. Replacement therapy involves the replacement of dead or dying cells with normal cells for restoring the function of a tissue or organ.

Several clinical trials of RPE transplantation for late AMD have demonstrated that improvement in visual function depends on the severity of the damage before RPE cell transplantation. The subsequent sections summarize current clinical trials of RPE transplantation.

#### 6.2.1. RPE Transplantation for Choroidal Neovascularization in AMD

Tezel et al. transplanted allogeneic RPE sheets obtained from cadavers after removing CNV lesions at the subfovea in AMD patients [129]. The patients received RPE cells from different cadavers, and no significant recovery in best-corrected visual acuity and contrast sensitivity was observed. Binder et al. transplanted RPE cells by subretinal injection of autologous RPE suspension after CNV excision at the subfovea in a prospective controlled trial [130]. One year after surgery, the best-corrected visual acuity and reading speed improved. Lu et al. also transplanted autologous RPE sheets in AMD patients after CNV excision [131] and reported recovery in best-corrected visual acuity. Kamao et al. and Nakagawa et al. transplanted autologous induced pluripotent stem cell-derived RPE sheets into the subretinal space in AMD patients after the surgical removal of subfoveal CNV [132,133,134]. Although the sheets were intact one year after surgery, no improvement was noted in the best-corrected visual acuity. Da Cruz et al. transplanted human embryonic stem cell-derived RPE in two AMD patients with subfoveal CNVs. One year after surgery, the best-corrected visual acuity and reading speed improved in only the patient with the least focal foveal atrophy [135]. Recently, the induced pluripotent stem cell (IPSc) based RPE is studied for transplantation [136,137,138]. It is expected to free from immune rejection, the most common adverse effects of transplantation, and showed well attached with AMD patients. However, their visual acuity was nearly unchanged [134]. 

#### 6.2.2. RPE Transplantation for GA in AMD

Schwartz et al. transplanted embryonic stem cell-derived RPE suspensions in nine AMD patients with GA [139,140]. The improvement in best-corrected visual acuity was 14 letters in eight eyes. Kashani et al. transplanted human embryonic stem cell-derived RPE monolayer sheets (3.5 mm × 6.25 mm) into the GA lesions in five AMD patients [141]. Four of the five patients presented no improvement in vision. However, the vision of the remaining patient improved by 17 ETDRS letters. 

## 7. Conclusions

Because AMD is a multifactorial disease with several pathways, a multifaceted intervention is required. Although the exact pathogenesis of AMD remains unknown, RPE dysfunction is a major contributor. Thus, several therapeutic strategies targeting RPE cells have been proposed. Among these, PBM is a promising noninvasive therapy to ameliorate oxidative stress, mitochondrial dysfunction, and complement dysfunction which are the main mechanisms of RPE dysfunction resulting in AMD. PBM can also remove drusen in AMD by activating RPE phagocytosis. Cell-based therapies involving transplantation of RPE sheets or cells are possible but will likely be beneficial only to patients with minimal photoreceptor atrophy in late AMD. Further research on the relationship between AMD pathogenesis and RPE dysfunction will identify other potential therapeutic targets.

## Data Availability

Not applicable.
